# The Preferred Directions of Conjunctive Grid X Head Direction Cells in the Medial Entorhinal Cortex Are Periodically Organized

**DOI:** 10.1371/journal.pone.0152041

**Published:** 2016-03-22

**Authors:** Alexander Thomas Keinath

**Affiliations:** Department of Psychology, University of Pennsylvania, Philadelphia, Pennsylvania, United States of America; University of Southern California, UNITED STATES

## Abstract

The discovery of speed-modulated grid, head direction, and conjunctive grid x head direction cells in the medial entorhinal cortex has led to the hypothesis that path integration, the updating of one’s spatial representation based on movement, may be carried out within this region. This hypothesis has been formalized by many computational models, including a class known as attractor network models. While many of these models propose specific mechanisms by which path integration might occur, predictions of these specific mechanisms have not been tested. Here I derive and test a key prediction of one attractor network path integration mechanism. Specifically, I first demonstrate that this mechanism predicts a periodic distribution of conjunctive cell preferred directions in order to minimize drift. Next, I test whether conjunctive cell preferred directions are in fact periodically organized. Results indicate that conjunctive cells are preferentially tuned to increments of 36°, consistent with drift minimization in this path integration mechanism. By contrast, no periodicity was observed in the preferred directions of either pure grid or pure head direction cells. These results provide the first neural evidence of a nonuniform structure in the directional preferences of any head direction representation found in the brain.

## Introduction

The ability to update one’s spatial representation based on movement–a process known as path integration–is crucial to the successful function of any allocentric spatial representational system. Since the discovery of speed-modulated grid, head direction, and conjunctive grid x head direction cells in the medial entorhinal cortex (mEC) [1–3], it has been widely hypothesized that this circuit might maintain path-integrated allocentric spatial representations, in the form of the grid code. This hypothesis has been formalized by a number of computational models, including a class known as attractor network models [4–7]. While attractor network models have generally been very successful at accounting for many properties of grid cells [8–10], predictions concerning proposed path integration mechanisms have not yet been tested. As such, whether and how path integration might occur in the mEC is not yet fully understood. To address this knowledge gap, I here derive and test one such prediction, the prediction that conjunctive cell directional preferences are periodically organized.

**Fig 1:**
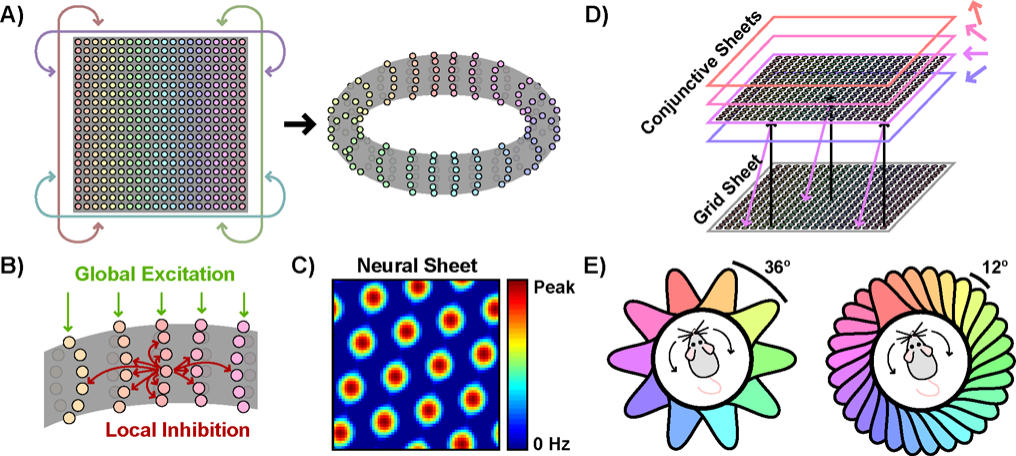
Constructing an attractor network model which implements a conjunctive cell path integration mechanism. a) A neural sheet with periodic boundaries is instantiated, which can be visualized as a torus. b) Neurons within the sheet uniformly inhibit neighbors within a certain radius. All neurons also receive uniform excitatory input. c) Due to the interaction of global excitatory input and local inhibition, this network can settle into a grid-like state of activity across the neural sheet. d) Each conjunctive sheet receives excitatory input from similarly-located neurons in the grid sheet. Furthermore, the activity of each conjunctive sheet is gained by both speed and angular distance from a particular preferred direction. The excitatory output projections from each conjunctive sheet to the grid sheet are shifted slightly forward relative to the preferred direction of that conjunctive sheet. Here, for ease of interpretation, these output projections align with the allocentric directional tuning of the conjunctive sheet. e) Two examples of possible symmetric distributions of preferred directions with different numbers of conjunctive sheets but the same directional tuning width.

This prediction stems from a particular hypothesized path integration mechanism in which grid activity is updated during movement via fixed inputs from speed-modulated conjunctive cells [4,7]. Deriving this prediction requires an overview of how this mechanism, and the attractor network models which implement this mechanism, function. In an example of one such model, a two-dimensional neural sheet with periodic boundaries, e.g. a torus, is instantiated (Fig 1A). Each neuron within this sheet uniformly inhibits all neighboring neurons within a certain radius; no connections are made to neurons beyond this radius (Fig 1B). When driven by some potentially uniform excitatory input, the network can settle into a stable grid-like pattern of activity (Fig 1C), reminiscent of the grid-like pattern of activity observed across space in the rate maps of grid cells.

In order to generate the grid-like spatial activity of single grid cells, however, the activity on this grid sheet must be translated appropriately as the navigator moves about the environment. This translation can be accomplished via populations of conjunctive cells. To do so, the model can be elaborated to include additional neural sheets, corresponding to conjunctive populations (Fig 1D). Each neuron in these conjunctive sheets receives excitatory input from similarly-located neurons in the grid sheet, allowing each conjunctive sheet to inherit the grid-like activity structure of the grid sheet. Furthermore, the activity of each conjunctive sheet is gained by both speed and angular distance from a particular preferred direction. Finally, each conjunctive sheet projections back to the grid sheet; however, these excitatory projections are shifted forward relative to the preferred direction of the particular conjunctive sheet. Thus during movement, the additional speed-modulated forward-projecting excitatory input from the appropriate conjunctive sheets can translate the grid sheet activity, maintaining a path-integrated allocentric spatial representation and giving rise to grid-like spatial activity at the single cell level.

For this conjunctive cell path integration mechanism to function properly, however, all directions of movement must result in an equal translation of the grid sheet activity. Because this mechanism relies on fixed inputs from speed-modulated conjunctive sheets to translate grid activity, achieving uniform translation requires that all directions of movement elicit equal magnitude responses. In other words, optimal (in the sense of accumulating minimal drift) path integration in this model requires minimizing the variability of the conjunctive response provoked across all directions of movement. Large differences in response magnitude across directions would result in greater translation during movement in over-represented directions, yielding a drifting and inconsistent grid representation over time.

Here, I address whether conjunctive cell directional tuning is consistent with the minimization of drift from this type of mechanism. I first demonstrate that a periodic distribution of conjunctive sheet preferred directions minimizes conjunctive response variability (Fig 1E). Deviations from periodicity consistently increase response variability, increases which cannot be compensated for by adjusting either tuning widths or the relative weighting of individual conjunctive sheets. Next I reanalyze existing datasets to test whether periodic structure can be observed in the distribution of conjunctive cell preferred directions. I find that, consistent with the minimization of drift from this mechanism, conjunctive cells are preferentially tuned to increments of 36°.

## Results

### A Periodic Distribution of Preferred Directions Minimizes Drift

**Fig 2:**
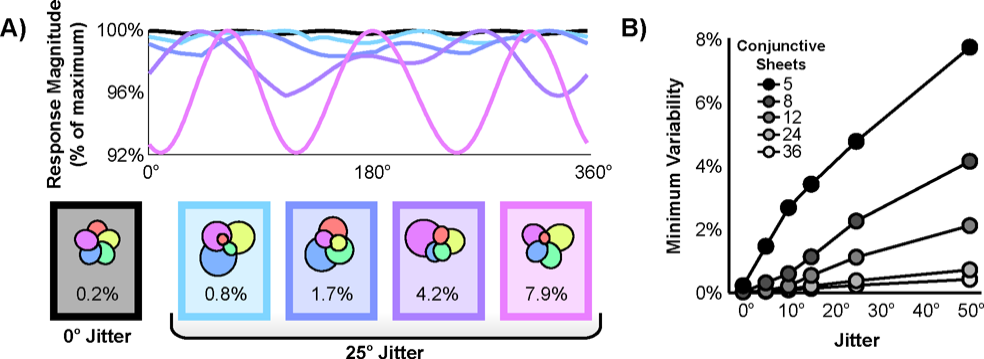
Deviation from periodicity increases conjunctive response variability. a) Response variability as a function of angle for five example simulations with five conjunctive sheets. Each polar plot depicts the tuning widths and relative weightings that minimized response variability given fixed jittered preferred directions. The minimum variability resulting from each simulation is indicated beneath each polar plot. b) Minimum variability as a function of both the number of conjunctive sheets and deviation from periodicity.

In attractor network models implementing this conjunctive mechanism, conjunctive response variability can be described as a function of 4 parameters: the number of conjunctive sheets, the distribution of preferred directions of these sheets, the directional tuning width of each sheet, and the relative weighting of the output of each conjunctive sheet. Intuitively, this response variability is closely related to the symmetry of the conjunctive sheet directional preferences (Fig 2A). As only a finite number of conjunctive sheets can be instantiated, a truly complete, infinitely-dense sampling of directions is unattainable. Thus, a symmetric distribution with conjunctive sheet preferred directions periodically tiling the space of all headings yields the closest approximation of a uniform response across directions. Deviations from a periodic distribution will then increase response variability, increases which may or may not be offset by readjusting either the tuning width or relative weighting of individual conjunctive sheets.

To formalize and test these intuitions, the minimum variability (and the drift it could accrue) was simulated as the distribution of preferred directions deviated more and more substantially from periodicity. To do so, first the preferred directions of the given *N* conjunctive sheets were evenly distributed at a period of 360°/*N*. Then, unique jitter with mean zero and a given standard deviation was added to each of these preferred directions. Finally, the tuning widths and relative weightings were adjusted to minimize response variability.

To measure response variability, the percent difference in response magnitude between the most over-represented direction and the most under-represented direction was computed. This value can be directly interpreted in terms of drift. Specifically, this value quantifies the difference in grid activity translation rate during journeys taken along the most over- and under-represented directions. For example, assuming this mechanism to be the only source of drift, a value of 2.5% indicates that 100 m journeys taken along the most over- and under-represented directions would yield an apparent discrepancy of 2.5 m in their resulting grid representations.

The effect of deviation from periodicity on the minimum variability for various numbers of conjunctive sheets is shown in Fig 2B. As one would expect, increasing the number of conjunctive sheets lowers the minimum variability; tiling the space with more Gaussians can more closely approximate a uniform distribution. Interestingly, however, even relatively small amounts of jitter lead to increases in the minimum variability, indicating that readjustments of individual tuning widths and relative weightings cannot fully compensate for deviations from periodicity. Although these values may seem small, they reflect drift on the order of >10 m that could accrue as a rat covers the large distances (100–1,000 m) typical of a day spent foraging. Thus, these results demonstrate that periodicity in the distribution of conjunctive sheet preferred directions is critically important for minimizing conjunctive response variability and the drift accrued by this mechanism.

### Conjunctive Cell Preferred Directions Are Periodically Organized

**Fig 3:**
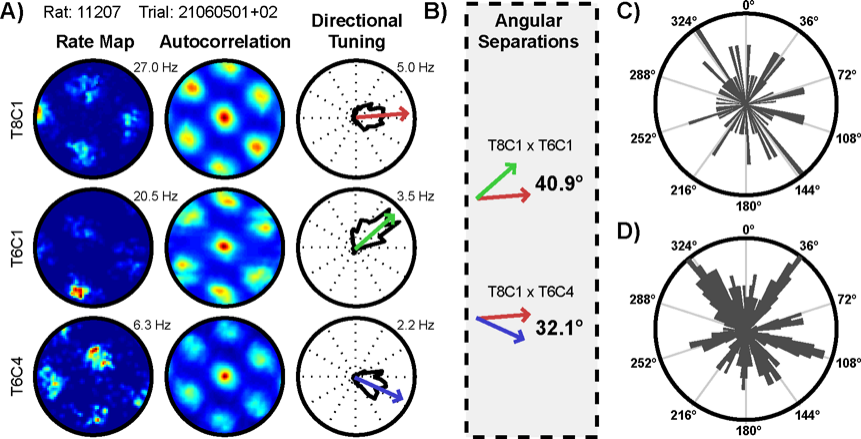
Examples of simultaneously recorded conjunctive cells, the resulting included angular separations, and the distribution of all conjunctive cell angular separations. a) Rate maps, autocorrelations, and directional tuning curves for the three simultaneously recorded conjunctive cells identified during this trial. Peak firing rates are indicated to the upper right of rate maps and directional tuning curves. b) The two included angular separations of the simultaneously recorded pairs of conjunctive cells for this same trial. c) A histogram of the raw distribution of all angular separations (3° bins, radius of 3 angles). d) The same histogram, smoothed with a 5 bin sliding average (3° bins, radius of 1.6 angles). Note that the peaks of this histogram align well with increments of 36°.

Is the distribution of conjunctive cell preferred directions periodically organized, consistent with optimal path integration carried out by this conjunctive mechanism? Data from [3], as well as data recorded prior to any experimental manipulations from [11] and [9], were reanalyzed to test this prediction. First a shuffling procedure was used to identify conjunctive cells which exhibited both significant gridness and significantly strong directional tuning. This procedure identified a total of 157 conjunctive cells in these datasets (Fig 3A). However, because the grid network may be aligned differently relative to the external reference frame across trials and rats, the preferred directions of individual conjunctive cells could not be directly compared across trials or rats with confidence. Therefore, rather than examining the preferred directions of individual conjunctive cells, further analysis focused on the angular separation of the preferred directions of simultaneously recorded conjunctive cell pairs (Fig 3B). Because angular separation is referenced to the network orientation, and not dependent on the network alignment with the external reference frame, angular separations can be compared across trials and rats with confidence. Furthermore, if conjunctive cell preferred directions are periodically organized, then the distribution of angular separations should also be periodically organized. Thus further analysis focused on the angular separation of 80 independent, simultaneously-recorded conjunctive cell pairs (14 rats; mean 5.7 angular separations per rat; range 1–31; S1 Fig). Histograms conveying the distribution of all angular separations are shown in Fig 3C and 3D.

Although some symmetric periodic structure in the distribution of angular separations is predicted, no particular period is a priori strongly motivated. To limit the number of prospective periods to a manageable number of statistical tests, practical and theoretical constraints were considered. Firstly, because the periodic distribution is hypothesized to be symmetric, only integer divisions of 360° were to be tested. Furthermore, as the predicted conjunctive sheets must permit translation in all directions on a 2D plane, at least 3 conjunctive sheets are necessary. This established a lower bound of 360°/3, or a period of 120°. Finally, if the noise in estimating the preferred directions exceeds the period separating these preferred directions, then any periodicity would be obscured. Such noise is further compounded by comparing the angular separation of conjunctive cell pairs. To estimate this noise, conjunctive cell preferred directions estimated from only odd minutes of data were compared to estimates from only even minutes of data. This yielded a median difference of 10.3°. Given this variability, a (likely overestimated) upper bound of 360°/36 was established, or a period of 10°. It should also be noted that positing a large divisor equates to positing large, and potentially implausible, numbers of conjunctive sheets. Based on these considerations, only 34 periods, corresponding to the set 360°/{3, 4, 5, …, 36}, were examined.

**Fig 4:**
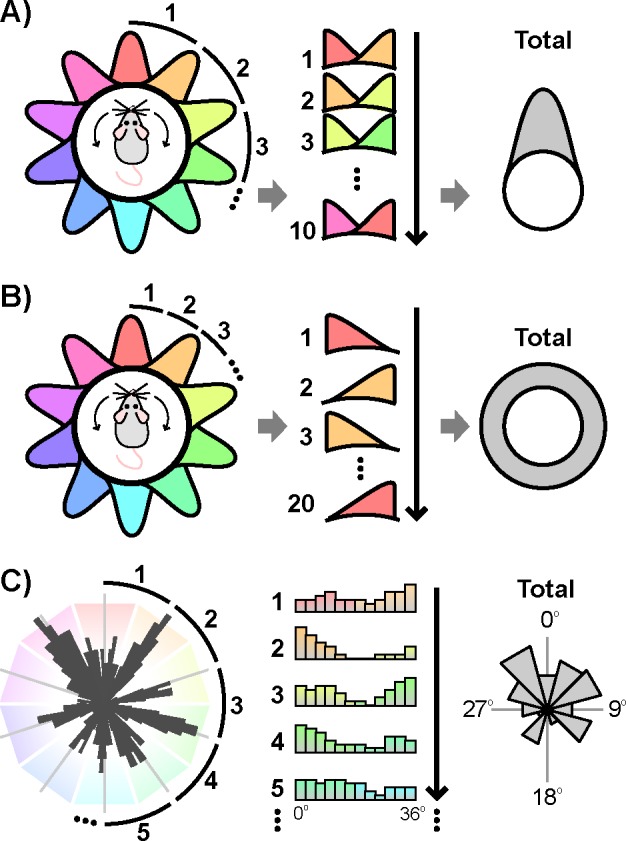
Testing for periodicity. a) If a circular periodic distribution is transformed by collapsing over partitions segmented by the proper period, the resulting circular distribution will be nonuniform and unimodal. b) If the same distribution is instead transformed by an improper period, the resulting circular distribution will approach uniformity. c) Schematic of this procedure performed on the set of angular separations with a period of 36° (smoothed distribution used for presentation purposes only).

The significance of periodic structure in the distribution of angular separations at each of these periods was then tested via the following logic. If a periodic circular distribution is transformed by collapsing over all partitions segmented by the proper period, then the resulting circular distribution will be nonuniform and unimodal (Fig 4A). If instead the chosen period does not match the periodicity of the distribution, then the resulting circular distribution should approach uniformity (Fig 4B). Thus the presence of a particular periodic organization can be tested by first collapsing over all partitions segmented at that period. Then standard circular statistics can assess the uniformity of this transformed distribution, with significant nonuniformity indicating periodic organization at the chosen period. In the current context, this procedure is analogous to first transforming the set of angular separations via the modulo operation with the chosen period as the modulus, and then testing the uniformity of the resulting transformed distribution. This method has an advantage over frequency-based approaches in that it is agnostic to the shape of the distribution.

**Fig 5:**
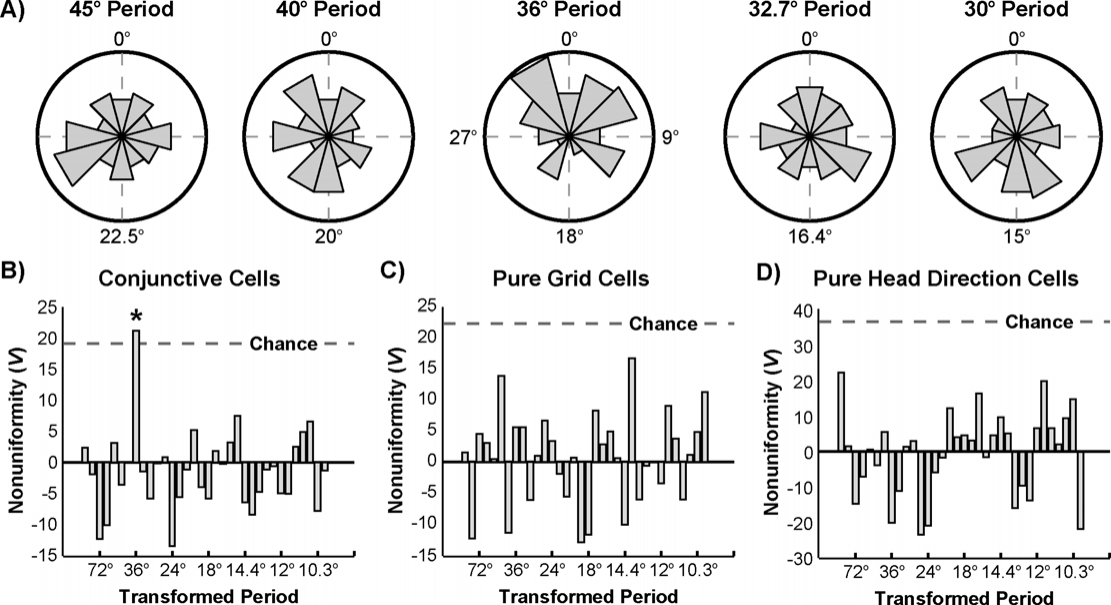
Periodicity in conjunctive cell preferred directions. a) Histograms of the resulting distributions of conjunctive cell angular separations when transformed via the modulo operation at each of the indicated periods (12 bins, radius of 13 angles). b) Kuiper’s *V* of the transformed distributions of conjunctive cell angular separations at each tested period. Dotted line indicates 0.05 significance criterion based on a nonparametric shuffling procedure which accounted for multiple comparisons. Note that only a transformation by a period of 36° yields a significantly nonuniform distribution. c) Analysis of pure grid cell angular separations. d) Analysis of pure head direction cell angular separations.

Accordingly, the set of angular separations was first transformed via the modulo operation at each of the tested periods (Fig 5A). Because the angular separations are referenced to the orientation of the network, periodic peaks in the distribution of angular separations are expected to occur at integer multiples of the period, with no additional phase offset. Thus, incorporating this phase offset prediction, the circular nonuniformity centered about 0° of each transformed distribution was quantified by Kuiper’s test. The test statistic for each of these tests is plotted in Fig 5B. The significance of each of these values was assessed via a nonparametric shuffling procedure that accounted for multiple comparisons. Only a single period of 36° achieved significance (p_corrected_ = 0.0178, *V* = 21.1). By contrast, when the same procedure was used to test for periodicity in the distribution of angular separations of pure grid (n = 111) and pure head direction (n = 301) cells, no period met significance (p_corrected_>0.7; Fig 5C and 5D). Together, these results indicate that the preferred directions of conjunctive cells are periodically organized, with conjunctive cells preferentially tuned to headings in increments of 36°.

## Discussion

Here I tested the prediction, derived from a hypothesized conjunctive cell path integration mechanism, that the distribution of conjunctive cell preferred directions is periodically organized. The results indicate that conjunctive cells are in fact preferentially tuned to headings in increments of 36°. These results are consistent with an attractor network model in which grid activity is updated during movement via fixed inputs from 10 conjunctive sheets while accruing minimal drift.

The prediction of periodic structure of conjunctive cell preferred directions put forth here is a product of this particular path integration mechanism. As such, this prediction may not be common to all attractor network models, or all computational models of grid cells more generally. For example, attractor network models which allow for plasticity in grid-conjunctive connections [5] or rely on different network architectures [12] may be more robust to deviations from periodicity. Likewise, conjunctive cells play a limited role in other classes of models, specifically oscillatory interference and self-organizing map models [13–17]. In these other classes of models, the grid-like structure observed in the rate maps of pure grid cells is generated without significant reliance on conjunctive cells. Thus, these classes of models do not strongly motivate any particular hypotheses about conjunctive cell directional tuning.

It is worth noting that the properties of grid, head direction, and conjunctive cells differ both along the dorsoventral axis and by cortical layer. In particular, both grid spacing–the distance between neighboring peaks of spatial activity–and directional tuning width generally increase when moving ventrally along this axis [18–20]. Furthermore, the increase in directional tuning width is layer-dependent: while directional tuning width increases along the dorsoventral axis in Layer III, tuning remains sharp in Layers V-VI [19]. Similarly, the periodic organization of conjunctive cell preferred directions may also differ along with grid spacing and tuning width, potentially in a layer-dependent manner. Unfortunately, the majority of conjunctive cells in these datasets were recorded from Layer III and exhibited similar grid spacing and tuning width, making it difficult to test this possibility directly here.

Lastly, the periodic structure observed here in conjunctive cell preferred directions differs markedly from what is known about pure head direction representations both within the mEC and in other regions, such as retrosplenial cortex [21,22], postsubiculum [23], lateral mammillary nuclei [24,25], and the anterodorsal thalamic nucleus [26]. In the current study and to the best of my knowledge in other studies examining pure head direction cells, the distribution of preferred directions either has not been or could not be significantly distinguished from uniformity [3,23,25,26]. This suggests that pure head direction representations, even those colocalized within the mEC, do not exhibit any of the low frequency periodicity observed here in the distribution of conjunctive cell preferred directions. Thus this low frequency periodicity, even divorced from the theoretical motivation highlighted here, may signify a major difference between the structure, function, and circuits underlying pure and conjunctive head direction representations.

## Methods

### Datasets

As this paper analyzes previously reported data, detailed behavioral, electrophysiological, and histological methods can be found in [3,9,11]. Briefly, neural activity was recorded from the mEC as male Long Evans rats foraged for randomly scattered vanilla or chocolate crumbs in familiar environments of different shapes and sizes: a small circular environment (90 cm diameter), a large circular environment (180 cm diameter), a small square environment (100 x 100 cm), or a large square environment (150 x 150 cm). Each environment contained an orienting cue card. Rats were tested for at least 10 min per trial. Only trials in which two LEDs were used to accurately track head direction were included in the current analysis.

### Simulating Response Variability

The variability in conjunctive response across directions was simulated as follows. The directional tuning of each conjunctive sheet was approximated by a wrapped circular Gaussian, with a preferred direction (mean) and tuning width (standard deviation). During one iteration, the preferred directions of all *N* conjunctive sheets were first evenly distributed at a period of 360°/*N*. Then, unique *iid* jitter with mean zero and a given standard deviation was added to each of these preferred directions. Next, a constrained optimization algorithm (Matlab’s *fmincon*) adjusted the tuning width and relative weighting of each conjunctive sheet to minimize response magnitude variability. Tuning width was initialized at 45° and constrained to the range [1°, 90°], with the maximum set to 90° to avoid minimizing variability by assigning arbitrarily large tuning widths. Relative weighting was initialized to uniformity, and constrained to be nonzero for each conjunctive sheet and sum to one across all conjunctive sheets. This procedure was repeated 1000 times for each level of conjunctive sheets and jitter standard deviation, with the mean response variability across these iterations taken as the measure of minimum variability. Response variability was numerically approximated by first sampling each conjunctive sheet tuning curve at 1° increments, then summing across all tuning curves to yield the overall response distribution. The final measure of conjunctive response variability was then the maximum of the overall response distribution minus its minimum, divided by its maximum.

### Rate Maps

Rate maps were created by first sorting the position into 3 x 3 cm^2^ bins, and creating two maps, one containing the number of spikes in each bin and the other the amount of time spent in each bin. Both maps were smoothed with a 2D isotropic Gaussian kernel with a standard deviation of 1 bin. The final spatial map was then created by dividing the spike map by the time map. Only pixels that were sampled for at least 0.2 s after smoothing were included as sampled.

### Autocorrelations

Autocorrelations of rate maps were computed as described in [3]. Briefly, overlapping bins of the original rate map and a shifted version of itself are correlated at a series of lags. To avoid artifacts due to few overlapping bins, only lags with an absolute value less than or equal to two-thirds the size of the rate map were included.

### Gridness

As in [3], gridness was computed by first correlating overlapping pixels of the autocorrelation with a rotated version of itself at 30°, 60°, 90°, 120°, and 150°. Gridness was then defined as the minimum of the correlations at 60° and 120° minus the maximum of the correlations at 30°, 90°, and 150°.

### Directional Tuning

To compute preferred firing directions and the strength of directional tuning for a cell, the heading data were first binned to intervals of 6°, and the average firing rate in each of these bins was calculated. No smoothing was applied. The circular average of these bins was then computed, with the angle of the resulting vector defining the preferred firing direction, and the length of the resulting vector defining the strength of the directional tuning. This method has the advantage that preferred firing direction is not discretized, as would occur if the bin with the highest firing rate were used to determine the preferred direction. Confirming this, binning by 5° or 8° did not result in any qualitative differences in results.

### Identification of Cell Types

A bootstrapping procedure was used to identify cells with significant gridness and significantly strong directional tuning. For both gridness and directional tuning, significance criteria were established by first circularly shifting the spiking activity of each cell relative to its position and heading data a random amount at least 30 s away from its actual alignment. The gridness and directional tuning strength of this shifted activity, which should no longer be correlated with position and heading, was then calculated for all cells, yielding distributions of both gridness and directional tuning strength with a matched number of cells. The 5^th^ percentiles of each of these distributions were then taken as this iteration’s estimate of significance criteria. This procedure was repeated 100 times, thus yielding distributions of significance criteria estimates. A cell was identified as significantly grid-like if its gridness met or exceeded the average gridness significance criterion (0.170 for these data). A cell was identified as significantly tuned to head direction if its directional tuning strength met or exceeded the average directional tuning strength significance criterion (0.151 for these data).

### Angular Separations

Angular separations were computed as the difference in angle of the preferred directions of pairs of simultaneously recorded conjunctive cells. For any session in which (*n*>1) conjunctive cells were identified, (*n*–1) angular separations were computed. Each cell contributed to at most 2 random angular separations, such that all angular separations were independent. For example, if (n = 3), then the angular separations (Cell 1 vs. Cell 2) and (Cell 2 vs. Cell 3) would be included. The angular separation (Cell 1 vs. Cell 3) would not be included, as its value is fully deducible from the other two included angular separations and therefore is not independent.

### Statistical Testing of Periodicity

The statistical significance of nonuniformity at each transformed period was assessed using a nonparametric shuffling procedure. The null distribution, corrected for multiple comparisons, was computed as follows. On a single iteration, the preferred directions contributing to all angular separations were first randomly reassigned to new pairs, and a new set of angular separations corresponding to these random cell pairs was computed. Next, Kuiper’s *V* was computed for each transformed period, and the maximum *V* across all periods was recorded. This process was repeated 100,000 times, yielding a null distribution of the maximum *V* one would expect by chance as a result of multiple comparisons. The 5^th^ percentile of this distribution was then selected as the criterion for evidence of significant periodicity corrected for multiple comparisons. The outcome of this procedure for conjunctive cell angular separations was a significance criterion of *V* = 19.2, slightly more strict than a Bonferroni correction. The full null distribution of maximum *V* was further used to estimate the reported corrected p-values. This procedure was repeated separately for pure grid (*V*_crit_ = 22.4) and pure head direction cells (*V*_crit_ = 36.9).

## Supporting Information

S1 Fig Angular Separations by Rat. Polar plots of the angular separations for each rat (black) plotted on increments of 36° (grey).
(TIF)
