# Supplementary figures and images for: The Preferred Directions of Conjunctive Grid X Head Direction Cells in the Medial Entorhinal Cortex Are Periodically Organized
Source: PLoS One. 2016 Mar 22;11(3):e0152041. doi: 10.1371/journal.pone.0152041 (PMC4803195; doi:10.1371/journal.pone.0152041)

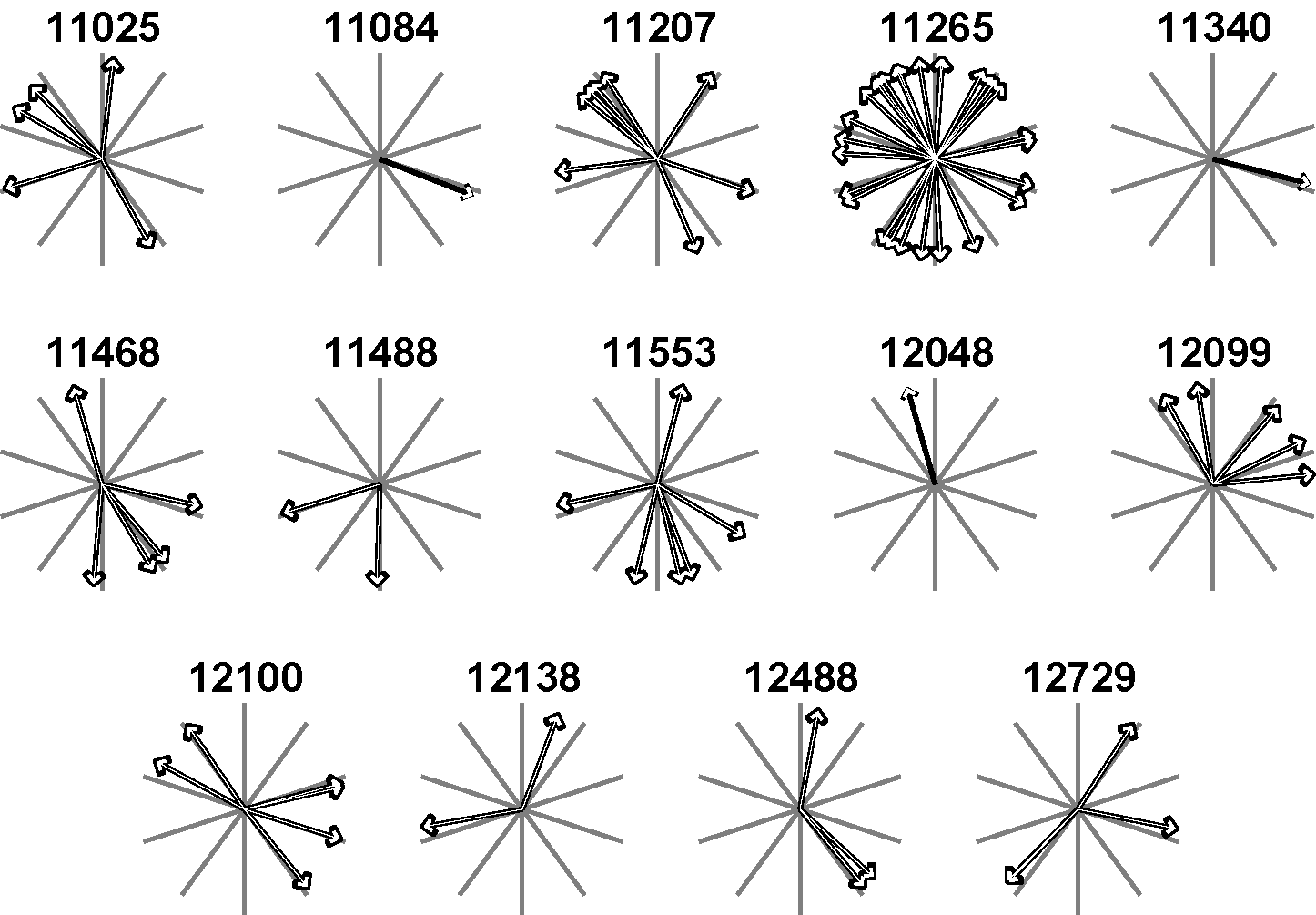

Supplement: S1 Fig — Polar plots of the angular separations for each rat (black) plotted on increments of 36° (grey). (TIF) [file pone.0152041.s001.tif]
